# Magnitude and direction of elbow torque asymmetries in manual wheelchair users

**DOI:** 10.3389/fspor.2023.1239626

**Published:** 2023-09-08

**Authors:** Grazieli Maria Biduski, Débora Aparecida Knihs, Silas Nery de Oliveira, Laís Peixoto Hoinaski, Mateus Rossato, Cíntia De La Rocha Freitas

**Affiliations:** ^1^ Laboratory of Biomechanics, Sport Center, Federal University of Santa Catarina, Florianópolis, Brazil; ^2^ Laboratory of Human Performance, Faculty of Physical Education and Physiotherapy, Federal University of Amazonas, Manaus, Brazil

**Keywords:** upper extremity, side-to-side difference, isokinetic torque, biomechanics, joint function

## Abstract

The aims of the present study are to investigate the magnitude and direction of the elbow torque asymmetries in manual wheelchair users and to verify the agreement levels of the asymmetry's direction between different velocities and contraction modes in the isokinetic test. The sample was composed of 14 manual wheelchair users (four women, 10 men). The peak torque of the elbow flexors and extensors were measured on the dominant and non-dominant limbs, using a set of concentric/eccentric contractions at speeds of 60° s^−1^ and 180° s^−1^. Asymmetries were calculated by a specific equation, and the levels of agreement of the asymmetry's direction were calculated using Kappa coefficient. The main results showed a large variability in the magnitude of the asymmetries, ranging from −73.1% (ND) to 59.9% (D) between participants. The agreement levels of the elbow flexors and extensors between the different contraction modes were great (*k* = 0.71–0.85) for most of the velocities [except for flexors of 60° s^−1^ (*k* = 0.29)], but the agreement levels were only slight to fair (*k* = 0.16–0.31) for most of the contraction modes when comparing between velocities [except for flexors eccentric (*k* = 0.71)]. In conclusion, the elbow torque asymmetries are highly variable between subjects in terms of magnitude. In addition, in general, the limb favored by the asymmetry is the same when comparing between velocities, but not when comparing between contraction modes.

## 1. Introduction

The manual wheelchair allows a person who is incapable of walking to have greater mobility and independence performing the functional tasks of their daily lives, as well as sports practice (1, 2). However, using a manual wheelchair, in everyday life, requires a great mechanical demand on the upper limbs (3, 4); and as a result of years of wheelchair propulsion, the users tend to have well-developed upper limbs (5).

Despite the fact that the shoulder joint receives the highest incidence of pain among wheelchair users (6), the articular complex of the elbow is, from a functional point of view, very important as well. Although not considered a load-bearing joint, the elbow supports a great amount of overload during the daily tasks performed by wheelchair users (7). It was observed that for the non-wheelchair population, the compression load on the elbows reaches 300 N, that is, 30.5 kg during simple activities such as eating and wearing clothes, and this overload can reach up to 1.730 kN (173 kg) when the body is supported on the arms (8). Carrying out suspensions, propelling, and even transfers in wheelchairs are common activities; thus, the elbow flexor and extensor muscles need to be strong.

Manual wheelchair propulsion is a repetitive and cyclical activity, in which the equal use of upper limbs is important to avoid overuse (9). Although this, in general, is considered a symmetric activity (9, 10), the preferred use of one limb (i.e., limb laterality) may produce imbalances between limbs by generating greater adaptations in one limb over the other (i.e., limb dominance), consequently leading to asymmetry appearance (11). In fact, asymmetries were verified in wheelchair propulsion (12), scapular kinematics (13), propulsion distance, power output, and speed (14) in manual wheelchair users. The literature suggests that force asymmetries between limbs are one of the problems that can cause muscle injuries (by the overuse of one limb) and consequently pain, which can limit movement and compromise the mobility of wheelchair users, taking them away from daily activities (15). In addition, reduced asymmetries are associated with better performance in upper limb sports, such as swimming (16). Thus, a balanced combination between limbs is desired.

Although some studies have evaluated upper limb strength during wheelchair handling (10, 12), the measurements obtained through isokinetic dynamometry have been considered the gold standard in determining the human joint function (e.g., torque/strength), being encouraged in studies involving Paralympic sports (17). So far, few studies had investigated strength asymmetries in wheelchair users (2, 9, 13), especially when considering isokinetic measures. The studies available in the literature had focused mainly on the magnitude of the asymmetry (i.e., asymmetry value) and analyzed the asymmetry as the group mean. Nonetheless, the individual analysis and the additional use of the direction of the asymmetry have been recently encouraged (18, 19). The asymmetry direction allows us to analyze which limb is favored by the asymmetry (i.e., dominance) (19).

The direction of the asymmetry can change over tasks or days of testing (18, 19), and investigating the agreement of the favored limb along different tests/days can be important for monitoring. When the magnitude of the asymmetry is analyzed alone, it can present a value favoring one limb during one test, and a similar value but favoring the contralateral limb in a subsequent test. If the direction of the asymmetry is not considered, it would appear that there were no changes in the athlete's asymmetry. In this context, both must be considered, the magnitude and direction of asymmetries, as a strategy for a clearer understanding of which changes are actually present (19). In the case of isokinetic dynamometry, tasks can be modified using different forms of muscle contraction and movement speeds, and to our knowledge, no studies have investigated the agreement level of the direction of the asymmetries between these variations.

As mentioned, the elbow flexors and extensors are important musculature for wheelchair users (7). In addition, asymmetries can have an impact on daily and sports activities of this population, by generating overuse or pain in one limb (15); thus, monitoring it seems relevant. While few studies were found investigating the magnitude of asymmetries in wheelchair users, to the best of our knowledge, no studies were found evaluating the individual direction of asymmetries in this population. We believe that considering both the magnitude of the asymmetries and direction may assist practitioners and coaches in conducting the training/treatments more assertively. Also, due to the task-dependency of asymmetries, the agreement levels of the asymmetry's direction between different kinds of torque testing on the isokinetic dynamometer need exploration as well. This can be helpful for choosing the tests to measure asymmetries. Thus, the aims of the present study were the following: (1) to investigate the magnitude and direction of the elbow torque asymmetries in manual wheelchair users, and (2) to verify the agreement levels of the asymmetry's direction between different velocities and contraction modes in the isokinetic test.

## 2. Materials and methods

### 2.1. Participants

A total of 14 manual wheelchair users (4 women, 32 ± 14.4 years; 46.5 ± 9.54 kg; 139 ± 30.9 cm, and 10 men, 37 ± 10.6 years; 76.8 ± 13.4 kg; 172 ± 9.67 cm) participated in the study. The participants’ characteristics are presented in Table 1. The eligibility criteria were as follows: (a) over 18 years old; (b) have a physical disability that prevents them from walking; and (c) using a manual wheelchair for over 1 year. Participants who had (a) metabolic disorders, (b) musculoskeletal injury (severe pain or movement limitation) in the joints of the upper limbs, and (c) medical restrictions for the practice of physical exercises were excluded from the data collection. The research project was previously approved by the Ethics and Research Committee on Humans (CAAE: n°15315219.0.0000.0121), and participants were informed about the procedures and signed an informed consent form, in accordance with the Declaration of Helsinki.

**Table 1:** Characteristics of study participants.

Participant	Sex	Age (years)	Time in MWC (years)	Physical deficiencies	Sports practice
1	F	30	12	SCI in T7 level	Surfing
2	M	41	19	SCI in C7 level	Hand cycling
3	F	53	30	SCI in T8 level	Surfing
4	M	27	10	SCI in T3/4 level	Handball
5	M	26	6	Osteogenesis	No
6	M	38	10	Myelomeningocele	Basketball
7	M	24	18	SCI in T7 level	Cycling
8	M	41	16	SCI in T12 level	Basketball
9	M	59	22	SCI in T12 level	Tennis
10	M	43	22	SCI in T12 level	No
11	M	32	7	SCI in T6/C7 level	Archery
12	M	45	8	Poliomyelitis	Basketball
13	F	28	24	Congenital Malformation	No
14	F	19	14	Myelomeningocele	Tennis
Mean	—	36.1 ± 11.5	15.5 ± 7.2	—	—

M, male; F, female; MWC, manual wheelchair; SCI, spinal cord injury; ASIA, American Spinal Injury Association impairment scale.

### 2.2. Anthropometric measures

Initially, the total body mass was assessed in a force platform (Kistler Quattro Jump, 9290 AD, Switzerland), where the wheelchair user was weighed in his/her own chair, and then the wheelchair mass was subtracted from the total mass, following the protocol utilized by Chen et al. (20). The height was self-reported to avoid embarrassments (21).

### 2.3. Torque evaluation

**Figure 1:**
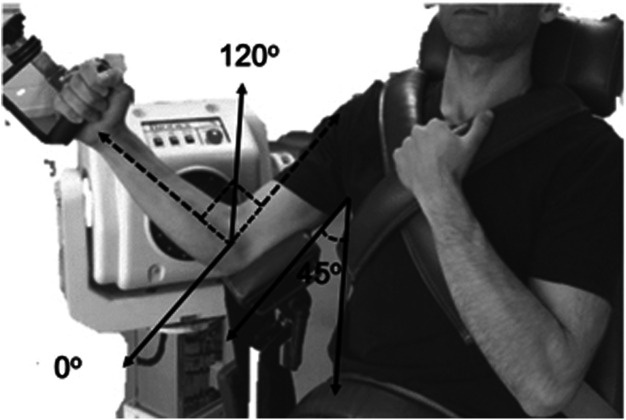
Positioning on the dynamometer.

The concentric/eccentric peak torque (PT) of both the flexors and extensors elbows muscles were assessed using an isokinetic dynamometer (Biodex System 4—Biodex Medical, USA). The isokinetic dynamometer was calibrated according to the manufacturer's instructions. The participants were positioned at the isokinetic dynamometer with the hips flexed at an angle of 85°, the shoulder was abducted at 45° to avoid compensatory movements of the shoulder joint (15). The range of motion of the elbow was 120°, where 0° represents the complete elbow extension (22). The volunteers were previously familiarized with the position on the dynamometer and the elbow flexion and extension movements that should be performed. The warm-up consisted of two sets of 10 elbow flexion and extension repetitions, using concentric strength at 120° s^−1^, with a passive recovery interval of 2 min between sets. The details about the setup data collection is presented in Figure 1.

The PT was evaluated by performing one set of four repetitions of concentric/eccentric contractions for the flexors and extensors elbow muscles at speeds of 60° s^−1^ and 180° s^−1^, and there was a 5-min rest interval between attempts. Verbal encouragement was given during all the tests. The limb (dominant or non-dominant) was randomly selected; however, the speed (60° s^−1^ or 180° s^−1^) and contraction mode were predefined.

### 2.4. Data analysis and variables

The PT values were extracted from the BIODEX and analyzed on the Python software (v.3). To determine the PT, the first contraction was disregarded, and the average of the other three contractions was calculated. The PT was evaluated in the dominant and non-dominant limbs, and the Waterloo questionnaire determined the dominance. The percentage difference between limbs (asymmetry values) was calculated through the Equation 1 (23). This is one of the most indicated equations for calculating asymmetries in unilateral tasks (23). This equation only provides positive values, utilized for absolute calculations and descriptive statistics analysis. To analyze the direction of the asymmetry, a negative sign was added when the asymmetry favored the non-dominant side.

(1) Asymmetrypercentage=100/(max_value)⋅(min_value)⋅(−1)+100

### 2.5. Statistical analysis

Initially, the descriptive statistics (mean and standard deviation) was calculated. Within session reliability was measured using the interclass correlation coefficient (ICC) with absolute agreement. The ICC scores were interpreted as >0.9 = excellent, 0.75–0.9 = good, 0.5–0.75 = moderate, and <0.5 = poor (24). Kappa coefficient was performed to analyze the agreement level of the asymmetry direction between velocities (60° s^−1^ vs. 180° s^−1^), and between contraction modes (concentric vs. eccentric). Kappa values were interpreted as 0.01–0.20 = slight, 0.21–0.40 = fair, 0.41–0.60 = moderate, 0.61–0.80 = substantial, and 0.81–0.99 = almost perfect (25). Statistical analysis was carried out on the SPSS v.17.0 (SPSS Inc., USA) software.

## 3. Results

The PT values, ICC, and mean asymmetry for both velocities and contraction modes of the elbow flexors and extensors are presented in Table 2. The ICC was classified as excellent for all conditions, with exception of the elbow flexors at 180° s^−1^, in eccentric contraction mode, for the non-dominant arm, in which the ICC was classified as good. The mean asymmetry values ranged from 15.4% (elbow flexors 180° s^−1^ concentric) to 22.6% (elbow extensors 180° s^−1^ concentric).

**Table 2:** Mean absolute peak torque values, ICC, and mean asymmetry for the elbow flexors and extensors muscles in both velocities and contraction modes.

Peak torque	Mean ± SD (N · m)	ICC (95% CI)	Mean asymmetry
EF 60° s^−1^ concentric D	35.5 ± 10.9	0.98 (0.95–0.99)	21.0% ± 17.3%
EF 60° s^−1^ concentric ND	34.2 ± 10.2	0.98 (0.96–0.99)	
EF 60° s^−1^ eccentric D	55.8 ± 17.6	0.95 (0.87–0.98)	21.9% ± 18.5%
EF 60° s^−1^ eccentric ND	57.8 ± 17.8	0.94 (0.77–0.98)	
EF 180° s^−1^ concentric D	31.3 ± 9.8	0.97 (0.92–0.99)	15.4% ± 13.3%
EF 180° s^−1^ concentric ND	32.2 ± 10.2	0.99 (0.98–0.99)	
EF 180° s^−1^ eccentric D	62.3 ± 22.6	0.93 (0.83–0.98)	17.7% ± 14.0%
EF 180° s^−1^ eccentric ND	54.7 ± 16.3	0.89 (0.71–0.96)	
EE 60° s^−1^ concentric D	36.9 ± 13.9	0.99 (0.97–0.99)	17.6% ± 14.4%
EE 60° s^−1^ concentric ND	38.9 ± 12.5	0.98 (0.95–0.99)	
EE 60° s^−1^ eccentric D	56.5 ± 22.6	0.99 (0.97–0.99)	19.8% ± 14.2%
EE 60° s^−1^ eccentric ND	59.2 ± 24.6	0.99 (0.96–0.99)	
EE 180° s^−1^ concentric D	27.8 ± 11.1	0.98 (0.96–0.99)	22.6% ± 17.6%
EE 180° s^−1^ concentric ND	27.9 ± 10.8	0.99 (0.97–0.99)	
EE 180° s^−1^ eccentric D	53.5 ± 19.3	0.98 (0.95–0.99)	16.8% ± 14.3%
EE 180° s^−1^ eccentric ND	53.7 ± 22.7	0.99 (0.97–0.99)	

EF, elbow flexors; EE, elbow extensors; D, dominant; ND, non-dominant; SD, standard deviation; ICC, interclass correlation coefficient; CI, confidence intervals.

**Figure 2:**
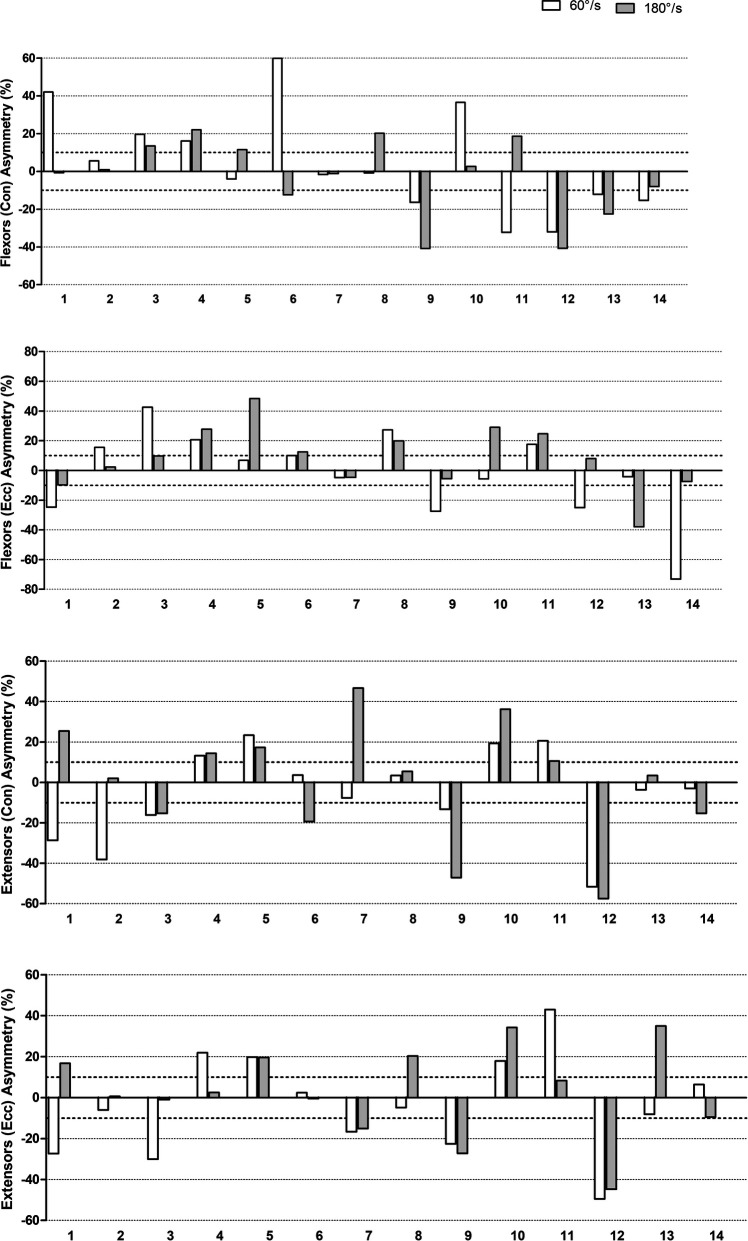
Individual asymmetry values for different velocities (60° vs. 180° s^−1^).

**Figure 3:**
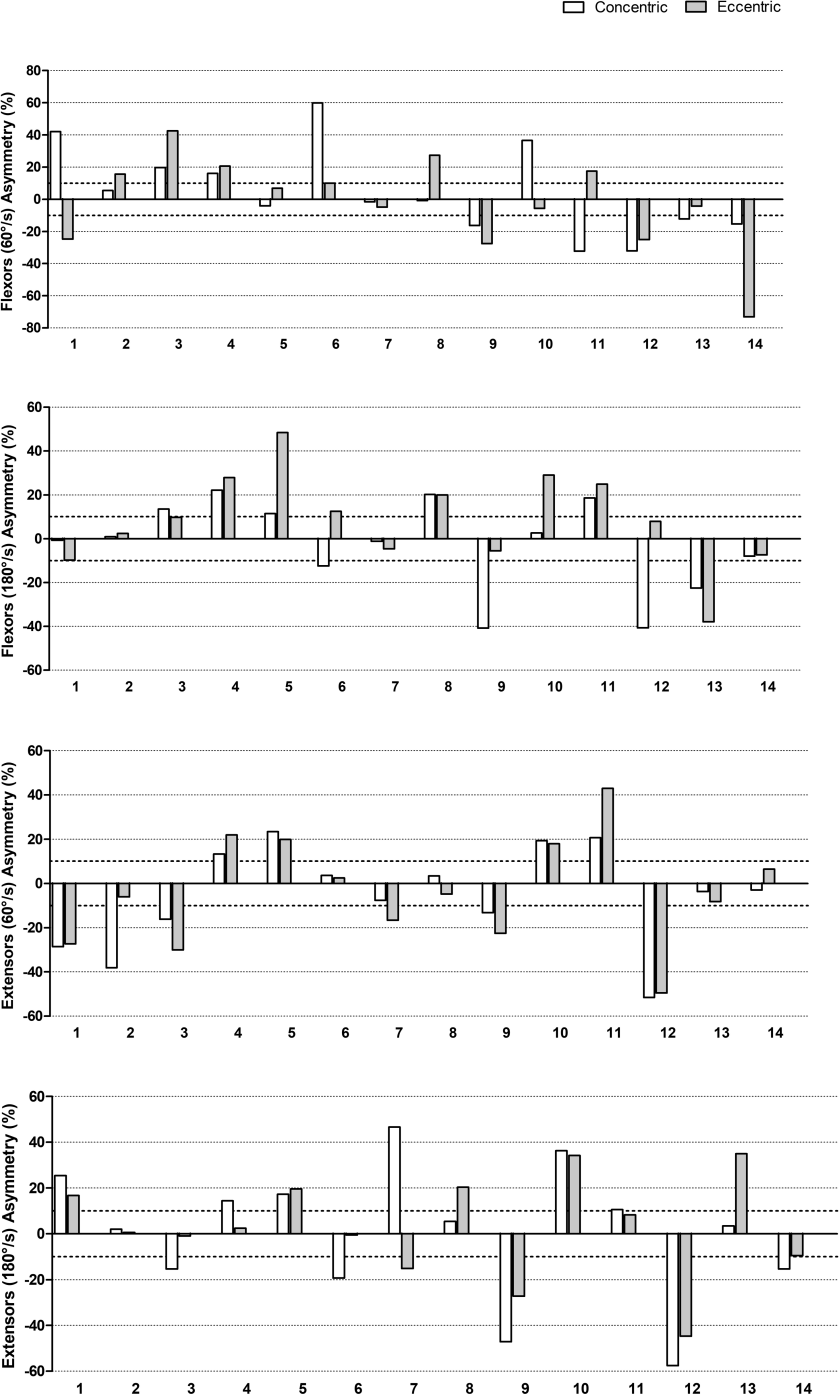
Individual asymmetry values for different contractions (concentric vs. eccentric).

Individual PT asymmetries (magnitude and direction) for the elbow flexors and extensors are graphically presented in Figure 2 (focus on different velocities) and in Figure 3 (focus on different contraction modes). As can be seen, there is a large variation in the asymmetry values between the participants, ranging from −73.1% to 59.9%. The figures also highlight the direction of the asymmetries, with negative values representing an asymmetry favoring the non-dominant side. A considerable variation on the asymmetry direction between participants can be observed as well.

The agreement level of the direction of asymmetry between the two tested velocities and the two contraction modes are presented in Tables 3, 4, respectively. For the two tested velocities, the asymmetry direction showed substantial levels of agreement (*k* = 0.71) only in the elbow flexors during eccentric contractions, pointing that the same side is favored by the asymmetry, independent of the velocity applied in the test. For the other contractions investigated, the direction of the asymmetries is quite variable between velocities (*k* = 0.16–0.31), that is, the side favored by the asymmetry in one velocity is not the same as in the other velocity.

**Table 3:** Kappa agreement level of asymmetry direction between different velocities.

	60° s^−1^ vs. 180° s^−1^	*P*	Classification
Flexors concentric	0.29	0.280	Fair
Flexors eccentric	0.71	0.005	Substantial
Extensors concentric	0.31	0.198	Fair
Extensors eccentric	0.16	0.533	Slight

**Table 4:** Kappa agreement level of asymmetry direction between different contractions.

	Concentric vs. eccentric	*P*	Classification
Flexors 60° s^−1^	0.29	0.280	Fair
Flexors 180° s^−1^	0.71	0.005	Substantial
Extensors 60° s^−1^	0.71	0.008	Substantial
Extensors 180° s^−1^	0.85	0.001	Almost perfect

When analyzing the agreement level of the direction of asymmetry between different contraction modes, the opposite was observed (Table 4). Great levels of agreement were demonstrated for the elbow extensors at both velocities and the elbow flexors at 180° s^−1^ (*k* = 0.71–0.85). Only the elbow flexors at 60° s^−1^ showed poor levels of agreement (*k* = 0.29). That suggests that for the elbow extensors, independent of velocity, and the elbow flexor at 180° s^−1^, the direction of the asymmetry is the same between the contraction modes.

## 4. Discussion

The present study aimed to investigate the magnitude and direction of the elbow PT asymmetries in manual wheelchair users and, in addition, to verify the agreement levels of the asymmetries direction between different velocities and contraction modes in the isokinetic test. The main results showed a large variability in the magnitude of the asymmetries, ranging from −73.1% to 59.9%, with a considerable variability in the direction of asymmetries between participants as well. In addition, the agreement levels of the elbow flexors and extensors between the different contraction modes were great for most of the velocities (except for flexors 60° s^−1^), but the agreement levels were only slight to fair for most of the contraction modes when comparing between velocities (except for flexors eccentric).

Regarding the PT magnitude of the asymmetries, the values were very distinct between participants, and the mean values, not considering the direction, were about 15.4%–22.6%, depending on the condition tested (contraction mode and velocity). To the authors’ knowledge, very few studies have measured the PT asymmetries of the elbow flexors and extensors using isokinetic tests in wheelchair users before. Moon et al. (2) investigated the shoulder and elbow strength asymmetries in male wheelchair tennis players, and verified significant side-to-side differences for all the conditions tested (flexion and extension, 60° s^−1^ and 180° s^−1^). The percentage asymmetry was not reported in the mentioned study, which precludes comparisons. It is important to highlight that the authors did not measure individual asymmetries or its direction. In the present study, due to the great variation in the magnitude of the asymmetry between participants, interpreting the results as a mean might mask some important information, so it is important to also consider the individual values presented in the Figures during the interpretation of the results.

Several studies have suggested a 10% cut-off value for side-to-side differences (23–25), and percentages above this would indicate risk to incidence of injury and/or performance losses. As can be observed in the individual results, several participants presented values greater than 10%, in at least one condition. However, this cut-off value has been considered arbitrary, since magnitude of the asymmetries can change depending on several factors (e.g., test, metric, determination equation) (19), making the use of a fixed value debatable. In this sense, the temporal follow-up using the same method for measurement and determination of the magnitude of the asymmetry is more relevant than a simple interpretation based on a fixed value. Practical interventions are suggested when high asymmetries values are observed, in order to prevent the overuse of one member.

The reasons for the big variation in the magnitude of asymmetries among the individuals in the present study can be varied, such as limb preference, arm dominance, injury history, and sports practice characteristics (11). In addition to the mentioned factors, the severity of spinal cord injuries should also be considered, where higher injuries tend to affect the functionality of the upper limbs. Specifically, regarding sports practice, as can be seen in Table 1, many participants practice sports with different between-arms demands, such as tennis, handball, basketball, and archery, while others perform symmetric sports such as surfing and hand cycling or do not practice any sport. Asymmetries can be a reflection of the sport-specific demand or a functional adaptation arising from the accentuated sports practice (11). However, there seems to be no pattern between the kind of sport practiced and the magnitude or direction of asymmetries, which once again makes evident the variable nature of asymmetries.

The direction of asymmetries was also varied when qualitatively compared between individuals. Many studies do not report the direction of the asymmetry, especially when the side-to-side differences are determined through conventional statistic tests (e.g., *t-*test). Using this parameter (i.e., direction) has grown along with using the individual analysis (18). Bishop et al. (18, 19) suggested that the magnitude and direction of asymmetries can change between different tests and variables. In addition, Boccia et al. (26) recently showed that strength asymmetries are also muscle-specific. This highlights the task-dependency of asymmetries (19, 27). Due to this task-dependency, in terms of comparison and/or monitoring, it seems important to have all information. For example, one individual performs a test, and the magnitude of the asymmetry of 13% is determined. On another occasion, a magnitude of −7% was obtained. If the direction of the asymmetry was not considered, the interpretation would be that the individual just lowered the asymmetry a little, when actually the limb that was favored by the asymmetry completely changed. For a clearer understanding of which changes really occurred, it is important to measure both the magnitude and direction of asymmetries (18).

Regarding the agreement levels of asymmetry direction, the findings suggest that the same limb is favored by the PT asymmetry in different contraction modes, but not in different velocities (with some exceptions), in the isokinetic test. Recently, Boccia et al. (26) verified low levels of agreement (*k* ≤ 0.16) for the asymmetry direction when comparing strength parameters (rate of force development and maximum voluntary force) between the flexors and extensors elbow muscles. As mentioned previously, the direction of the asymmetries can vary between conditions, and that has some practical implications when testing and monitoring the asymmetries. From a practical point of view, the results of the present study advertise comparisons between elbow PT asymmetries when using isokinetic tests performed in different velocities, but also depending on the muscle group. On the other hand, it seems that there will be a considerable consistency in the limb favored by the elbow PT asymmetry if comparisons between different contraction modes were performed. Researchers and coaches should take this into account when testing and interpreting the asymmetries.

The sum of the results highlights the need for a specific and individual analysis of asymmetries for this population, as for others. If the results were analyzed just as a mean, some individuals would have their asymmetry values underestimated, while others would be overrated. Not to mention that, when the values are reported only as a mean, it is not possible to report the direction of the asymmetries. Taking into consideration that when an asymmetry is present, one limb is being more requested than the other, and our results are important to show that, even with a high variation, asymmetries are seen in wheelchair users, which may demand attention. In addition, it was already known that asymmetries could vary between tests, but our results showed that even in the same test, asymmetries can behave differently in terms of which limb is favored, depending on the protocol. This is important when considering which test protocol should be chosen to measure asymmetries, especially in a longitudinal or comparative perspective. Finally, using the asymmetry measurements of individual magnitude and direction may assist practitioners and coaches in carrying out a more specialized training intervention, which can help reducing asymmetries when necessary.

The present study has limitations that should be taken into consideration for a better interpretation of the results. First, the sample size is relatively small, and the population is very specific. Thus, the results should be replicated in other populations, with larger sample sizes, before extrapolation. On the other hand, this reinforces the need for an individual analysis. A re-test session was not conducted. This does not allow knowing whether the results would replicate on other testing days, which could be an interesting question to be answered in future research. Different and more functional tests could also bring interesting answers to the topic for this population, with new studies being encouraged.

## 5. Conclusions

From the results of the present study, it can be concluded that elbow PT asymmetries are highly variable between wheelchair users in terms of magnitude and direction. In addition, the limb favored by the asymmetry is the same when comparing between velocities, but not when comparing between contraction modes, which is important when choosing a testing protocol. The finding highlights the need for an individual analysis of asymmetries, especially when seeking for a temporal monitoring.

## Data Availability

The raw data supporting the conclusions of this article will be made available by the authors, without undue reservation.
